# Isolation of a Novel Fusogenic Orthoreovirus from *Eucampsipoda africana* Bat Flies in South Africa

**DOI:** 10.3390/v8030065

**Published:** 2016-02-29

**Authors:** Petrus Jansen van Vuren, Michael Wiley, Gustavo Palacios, Nadia Storm, Stewart McCulloch, Wanda Markotter, Monica Birkhead, Alan Kemp, Janusz T. Paweska

**Affiliations:** 1 Centre for Emerging and Zoonotic Diseases, National Institute for Communicable Diseases, National Health Laboratory Service, Sandringham 2131, South Africa; petrusv@nicd.ac.za (P.J.v.V.); nadias@nicd.ac.za (N.S.); monicab@nicd.ac.za (M.B.); alank@nicd.ac.za (A.K.); 2 Department of Microbiology and Plant Pathology, Faculty of Natural and Agricultural Science, University of Pretoria, Pretoria 0028, South Africa; Stewart.Mcculloch@up.ac.za (S.M.); Wanda.Markotter@up.ac.za (W.K.); 3 Center for Genomic Science, United States Army Medical Research Institute of Infectious Diseases, Frederick, MD 21702, USA; michael.r.wiley19.ctr@mail.mil (M.W.); gustavo.f.palacios.ctr@mail.mil (G.P.); 4 Faculty of Health Sciences, University of the Witwatersrand, Johannesburg 2193, South Africa

**Keywords:** *Rousettus aegyptiacus*, fusogenic orthoreovirus, *Reoviridae*, *Nycteribiidae*, bat flies, *Eucampsipoda africana*

## Abstract

We report on the isolation of a novel fusogenic orthoreovirus from bat flies (*Eucampsipoda africana*) associated with Egyptian fruit bats (*Rousettus aegyptiacus*) collected in South Africa. Complete sequences of the ten dsRNA genome segments of the virus, tentatively named Mahlapitsi virus (MAHLV), were determined. Phylogenetic analysis places this virus into a distinct clade with Baboon orthoreovirus, Bush viper reovirus and the bat-associated Broome virus. All genome segments of MAHLV contain a 5' terminal sequence (5'-GGUCA) that is unique to all currently described viruses of the genus. The smallest genome segment is bicistronic encoding for a 14 kDa protein similar to p14 membrane fusion protein of Bush viper reovirus and an 18 kDa protein similar to p16 non-structural protein of Baboon orthoreovirus. This is the first report on isolation of an orthoreovirus from an arthropod host associated with bats, and phylogenetic and sequence data suggests that MAHLV constitutes a new species within the *Orthoreovirus* genus.

## 1. Introduction

Bats have been increasingly associated with emerging and re-emerging viruses. The likelihood of possible transmission of these pathogens to humans is ever increasing as a result of human encroachment on animal habitats, climate change and change of human behaviour. Pathogens of particular public health importance are filoviruses [1,2], coronaviruses [3,4], paramyxoviruses [5,6] and lyssaviruses [7,8]. Other viruses, without a known human disease link, have also been detected recently [9,10,11]. Some human pathogens, such as Rift Valley fever virus, that have been detected in bats were likely a result of coincidental infection and do not constitute proof that bats play a role as reservoirs [12].

Bats are parasitized by a number of ectoparasites, including mites, bat flies, ticks and fleas, and often by some or all of these simultaneously [13]. The bat flies are members of two families in the Diptera order, namely, the *Streblidae* and *Nycteribiidae*, and are highly host-specific obligate ectoparasites of bats [13,14,15]. Both bat fly families are hematophagous and potentially capable of mechanical or biological pathogen transmission. However, to date only *Bartonella spp*. [16,17] and viruses from the *Rhabdoviridae* family [18] have been detected in bat flies.

*Reoviridae* is a large and diverse family comprised of non-enveloped viruses containing segmented, double stranded (ds) RNA genomes and consists of two sub-families, *Sedoreovirinae* and *Spinareovirinae* [19]. The *Orthoreovirus* genus is in the *Spinareovirinae* sub-family and consists of five formally recognized species: *Avian orthoreovirus*, *Baboon orthoreovirus*, *Nelson bay orthoreovirus,* *Reptilian orthoreovirus* and *Mammalian orthoreovirus,* the type species. The *Nelson Bay* species group consists of viruses isolated from or associated with flying foxes [20,21,22,23], whilst several mammalian orthoreoviruses have also been isolated from bats [24,25]. *Broome virus*, a fusogenic virus isolated from a flying fox, has been proposed as the sixth orthoreovirus species based on its sequence divergence from other viruses in the genus [26]. Another proposed new species, W*ild bird orthoreovirus*, consists of three viruses that form a distinct clade from other avian orthoreoviruses [27]. A reovirus has been isolated from an aborted Stellar sea lion that groups in a clade with avian reoviruses and Nelson bay orthoreovirus [28]. Orthoreovirus infection in different hosts results in a variety of disease symptoms, including arthritis, pneumonia, neurological disease and acute respiratory illness [22,23,29,30,31]. Some bat-associated viruses in the *Orthoreovirus* genus, such as Melaka, Kampar and Nelson Bay orthoreoviruses, have been linked to human disease, usually of a respiratory nature and capable of limited human-to-human transmission [22,23,32,33]. Orthoreoviruses are thought to only infect vertebrates, based on the current known host range for the genus.

One aspect of bat-borne pathogen transmission that has been largely overlooked is the role of hematophagous arthropod ectoparasites of bats. This complexity is compounded by the fact that the ectoparasites themselves are not well studied and little is known about their life cycles.

Here we report the first isolation and characterization of an orthoreovirus from arthropod ectoparasites of the Egyptian fruit bat (*Rousettus aegyptiacus*). We determined the complete sequences [34] of all ten genome segments and propose that it be regarded as a new species within the *Orthoreovirus* genus of the *Reoviridae* family based on its phylogeny and unique sequence characteristics, as well as the apparent unique host and ecological niche.

## 2. Materials and Methods

### 2.1. Ethics and Permit Statement

This study was carried out in strict accordance with the recommendations of the South African National Standards for the Care and Use of Animals for Scientific Purposes (SANS 10386:2008). The protocols for field sampling and transport of *Rousettus aegyptiacus* and samples collected from this species is approved by the National Health Laboratory Service Animal Ethics Committee (AEC 137/12), University of Pretoria Animal ethics committee (EC054-14), Department of Economic Development, Environment & Tourism: Limpopo Province Directorate: Wildlife Trade and Regulation Permit (CPM 006806) and the South African Department of Agriculture, Forestry and Fisheries (Section 20 approval 12/11/1/1/8).

### 2.2. Sample Collection and Processing

**Figure 1:**
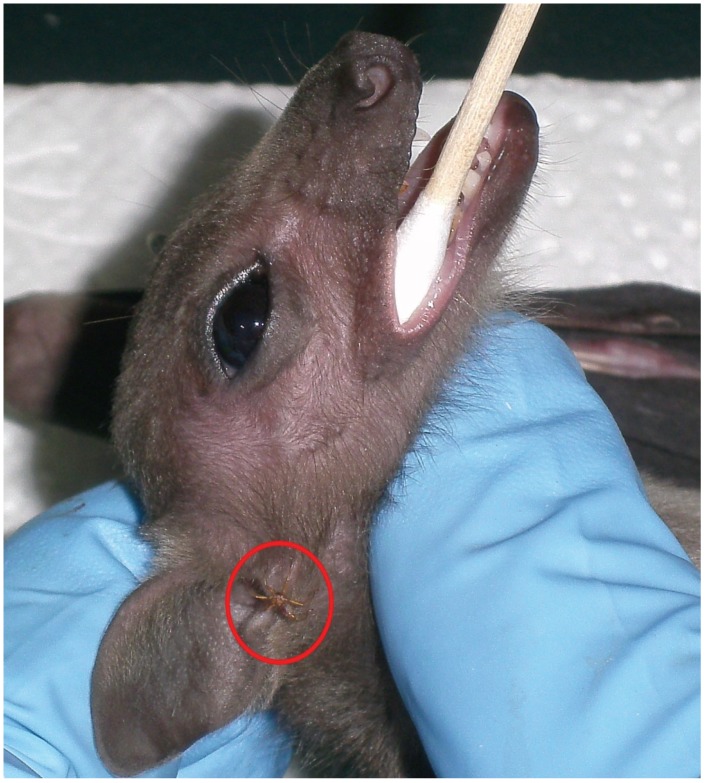
Bat fly (family *Nycteribiidae,* species *Eucampsipoda africana*) at the ear lobe of an Egyptian fruit bat undergoing sampling. The fly is indicated by the red circle.

*Rousettus aegyptiacus* bats were sampled on a monthly basis from March 2013 until March 2014 at Mahune Cave in the Mahlapitsi Valley, Limpopo province, South Africa, using standard trapping procedures [35] as part of a surveillance project of zoonotic pathogens harboured by South African bats. Ectoparasites were collected from the bats (Figure 1) into cryotubes containing 0.5 mL Dulbecco’s modified Eagle’s medium (DMEM, Lonza, Basel, Switzerland). Samples were transferred to vapour phase liquid nitrogen field storage and transported back to the biosafety level four laboratory (BSL4) at the National Institute for Communicable Diseases (NICD) in Johannesburg for further processing. Bat flies from individual bats were pooled into a single tube per bat. Pools were homogenized at 30 Hz for eight minutes by using a Tissuelyzer II (Qiagen, Hilden, Germany) and 5mm stainless steel beads (Qiagen). Cellular debris was removed by centrifugation at 14,000× *g* for 3 min, and the supernatant used for subsequent virus isolation and nucleic acid extraction procedures.

### 2.3. Virus Isolation and Titration

The wells of 24-well tissue culture plates (Nunc) were seeded with Vero E6 cells and grown to 80%–90% confluency in Eagle’s minimum essential medium (EMEM, Lonza) supplemented with antibiotics (Penicillin/Streptomycin/AmphotericinB, Lonza) and 10% foetal calf serum at 37 °C and 5% CO_2_. Culture medium was removed and the monolayers in individual wells inoculated with 200 µL of ectoparasite pool homogenates (one pool representing parasites from one bat). After one hour adsorption at 37 °C, the inoculum was removed and fresh EMEM containing antibiotics and 2% foetal calf serum added. The 24-well plates were incubated for 14 days and cytopathic effects (CPE) monitored. A second and third blind passage was performed for all samples by inoculating and incubating monolayers as described above with 200 µL of undiluted supernatant from the preceding passage. Supernatants were collected from all wells displaying CPE after three blind passages, a 1/10 dilution prepared in EMEM, and 1 mL of this used to inoculate a 25 cm^2^ tissue culture flask. If the same CPE was noted in the sub-cultured 25 cm^2^ flask, a 1/100 dilution of this supernatant was prepared and used to inoculate a 75 cm^2^ tissue culture flask for preparation of stock virus.

Stock virus titres were determined by standard tissue culture infectious dose 50 (TCID_50_) titrations on 96-well microtitre plates as described previously [36].

### 2.4. Transmission Electron Microscopy (TEM)

For electron microscopy specimen preparation, 80%–90% confluent Vero E6 monolayers in 25 cm^2^ flasks were inoculated with stock virus and monitored for CPE. At the first sign of CPE, culture supernatant was collected, cleared of cellular content by centrifugation (3000× *g* for 5 min), and subsequently fixed in an equal volume of 2.5% glutaraldehyde in 0.1 M Hepes buffer (pH 6.9) for visualization of virus particles by negative staining. A Beckman Airfuge^®^ (Beckman Coulter, Brea, CA, USA) was used to concentrate all samples (10 min at 207 kPa), after which droplets of sample were adsorbed to 0.25% formar-coated copper grids for a minimum of 10 min, rinsed twice in deionised, distilled water and stained briefly in 2% phosphotungstic acid (pH 6.9). For ultramicrotomy, the remaining infected monolayers were flooded with the same fixative overnight, then routinely processed (postfixation in 1% buffered osmium tetroxide, graded ethanol dehydration, infiltration with a low viscosity resin (Agar Scientific, Stansted, UK) and overnight polymerisation at 70 °C). Seventy nm sections were cut on a Leica EM-UC6, double stained with saturated uranyl acetate and lead citrate, and viewed at 80 kV on a BioTwin Spirit (FEI Company, Hillsboro, OR, USA). Imaging was done with an Olympus Quemesa CCD camera (Olympus, Tokyo, Japan).

### 2.5. Sequence-Independent Single-Primer Amplification (SISPA), Rapid Amplification of cDNA Ends (RACE), Next-Generation Sequencing (NGS) and Bioinformatics

Stock virus culture supernatant was added to Trizol-LS (Life Technologies, Waltham, MA, USA) at a ratio of 100 µL supernatant to 300 µL Trizol-LS. RNA was extracted using a column based kit (Direct-Zol RNA kit, Zymo Research, Irvine, CA, USA). To increase sensitivity, rRNA was depleted using the same method as described previously [37]. RNAs were converted to cDNA and amplified using SISPA as described previously with modifications [38]. To enhance coverage of the terminal ends, an oligo containing three rGTP at the 3' end (GCCGGAGCTCTGCAGATATCGGCCATTATGGCCrGrGrG) was added during first-strand cDNA synthesis and the reverse transcriptase was changed to Maxima H Minus (Thermo Scientific, Waltham, MA, USA), which has terminal transferase activity that enables addition of the rGTP containing oligo to the 5' end during cDNA synthesis. Amplicons were sheared and libraries prepared using the Illumina TRuSeq DNA Library preparation kit (Illumina, San Diego, CA, USA). Sequencing was performed either on an Illumina MiSeq (Illumina) or NextSeq 500 (Illumina) using either a 2 × 150 or 2 × 250 version2 kit. Illumina and SISPA adapter sequences were trimmed from the sequencing reads using Cutadapt-1.2.1 [39], quality filtering was conducted with Prinseq-lite (-min len 50-derep 14-lc method dust-lc threshold 3-trim ns left 1-trim ns right 1-trim qual right 15) [40] and reads were assembled into contigs using Ray Meta with kmer length = 25 [41]. Resultant contigs were aligned to the NCBI sequence database using BLAST.

### 2.6. Virus Phylogenetic, Genome and Protein Sequence Analysis

The MEGA (version 6) program was used to prepare alignments (ClustalW) of nucleic acid segment sequences, deduced amino acid sequences, phylogenetic trees and pairwise distance calculations [42]. The publicly available reovirus sequences used in the analysis were obtained from NCBI-Nucleotide (Genbank). Nucleotide sequences from a small number of viruses from each genus in the *Reoviridae* family were used to prepare a Maximum Likelihood tree showing the placement of MAHLV in the family based on the full RNA-dependent RNA polymerase (RdRp) encoding segment. Maximum Likelihood trees were prepared using amino acid sequences of all open reading frames from all segments, showing the placement of Mahlapitsi virus (MAHLV) in the *Orthoreovirus* genus relative to other viruses in this genus for which sequence is available on Genbank. Virus sequence accession numbers are summarised in Table 1. The evolutionary history was inferred by using the Maximum Likelihood method based on the JTT matrix-based model [43]. The tree with the highest log likelihood is shown. The percentage of trees in which the associated taxa clustered together is shown next to the branches (1000 bootstrap iterations). Initial tree(s) for the heuristic search were obtained by applying the Neighbor-Joining method to a matrix of pairwise distances estimated using a JTT model. The tree is drawn to scale, with branch lengths measured in the number of substitutions per site All positions containing gaps and missing data were eliminated. Evolutionary analyses were conducted in MEGA6 [42]. Open reading frames were located and deduced protein amino acid sequences prepared by using the CLC Genomics Workbench (Qiagen). Putative functions of the new virus deduced proteins were determined by BLASTx similarity searches to sequences available on Genbank.

### 2.7. Phylogenetic Analysis of the Bat Fly

The ectoparasite pool homogenate used for virus isolation was used as DNA source for phylogenetic confirmation of species. DNA was extracted using Trizol (Invitrogen, Waltham, MA, USA) and the method as described by the manufacturer. Amplification of the cytochrome c oxidase subunit I (COI) gene was performed with barcoding primers as described by Tortosa *et al*.: LCO1490 and HCO2198 [13]. Polymerase chain reaction was carried out in 50 µL reactions containing 25 µL MyTaq Red mix 2× (Bioline, London, UK), 2 µL of forward and reverse primer (10 µM), DNA template (10 µL) and nuclease free water (11 µL). Amplification steps were 94 °C for 5 min, 25 cycles of 94 °C for 60 s, 48 °C for 60 s and 72 °C for 90 s, and 72 °C for 10 min. PCR product was purified using the MinElute kit (Qiagen). Purified PCR amplicon products were then sequenced at the NICD Core Sequencing Facility (NICD, Sandringham, South Africa).

### 2.8. Virus Growth Curves

Replication of MAHLV was evaluated in two cell lines: Vero E6 (source African green monkey kidney) and C6-36 (source *Aedes albopictus* mosquitoes). Cells were grown to 50%–70% confluency in 25 cm^2^ flasks, supernatant removed and respective flasks inoculated with 1 mL of 10^−1^, 10^−2^, 10^−3^ and 10^−5^ dilutions from stock virus (1 × 10^6^ TCID_50_/mL) in EMEM. After 1 h adsorption at 37 °C (VeroE6) or 28 °C (C6-36), the inoculum was removed, cells washed with 5 mL phosphate buffered saline (PBS) and fresh EMEM, antibiotics and 2% foetal calf serum (Hyclone, Logan, UT, USA) added. Cultures were incubated for 13 days at 37 °C (VeroE6) or 28 °C (C6-36) while 0.5 mL aliquots of supernatant were collected from each flask directly after inoculation and addition of fresh medium (day 0), followed by day 4, 7 and 13. RNA was extracted from 140 µL of the serial supernatant collections (QIamp viral RNA kit, Qiagen) and subjected to TaqMan real-time RT-PCR. A Taqman real-time RT-PCR was developed to detect the RdRp gene of MAHLV. Primers and probe sequences are: forward MORV_796F (5'-TAGTGGTTCGTATGCGTGGT-3'), reverse MORV_893R (5'-AACAGCCATTCAATCTCAGG-3') and probe MORV_875P (*FAM*-GGCACATATCCCTCAACTGG-*BHQ*), with the number in the oligonucleotide name indicating the nucleic acid position in the segment encoding RdRp. Real-time RT-PCR was performed on the extracted RNA using the Qiagen One-step RT-PCR kit (Qiagen) on a SmartCycler (Cepheid, Sunnyvale, CA, USA) with the following program: reverse transcription (50 °C for 30 min), hot-start Taq activation (95 °C for 15 min) and 50 cycles of amplification (95 °C for 15 s; 52 °C for 25 s plus signal acquisition; 72 °C for 20 s). RNA extracted from diluted stock MAHLV (final 1 × 10^5^ TCID_50_/mL) was used as a qualitative positive control in each run.

## 3. Results

### 3.1. Isolation of a Syncytia-Forming Virus from Bat Ectoparasite Pools

**Figure 2:**
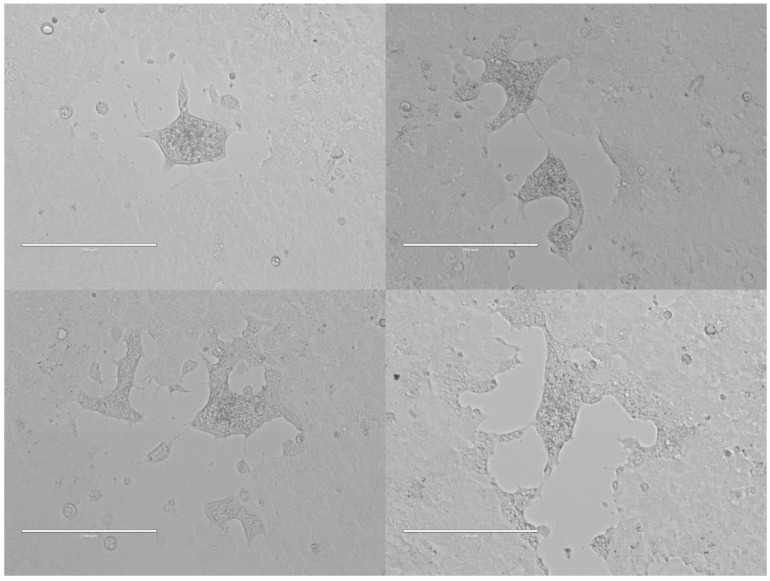
Cytopathic effects in the form of syncytia formation cause by MAHL (Mahlapitsi virus) in Vero E6 cells, from four days post infection onwards.

**Figure 3:**
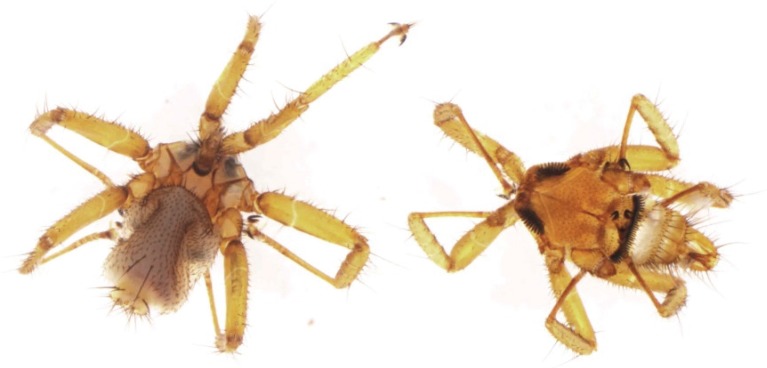
*Eucampsipoda africana* collected from an Egyptian fruit bat captured in Limpopo Province, South Africa.

**Figure 4:**
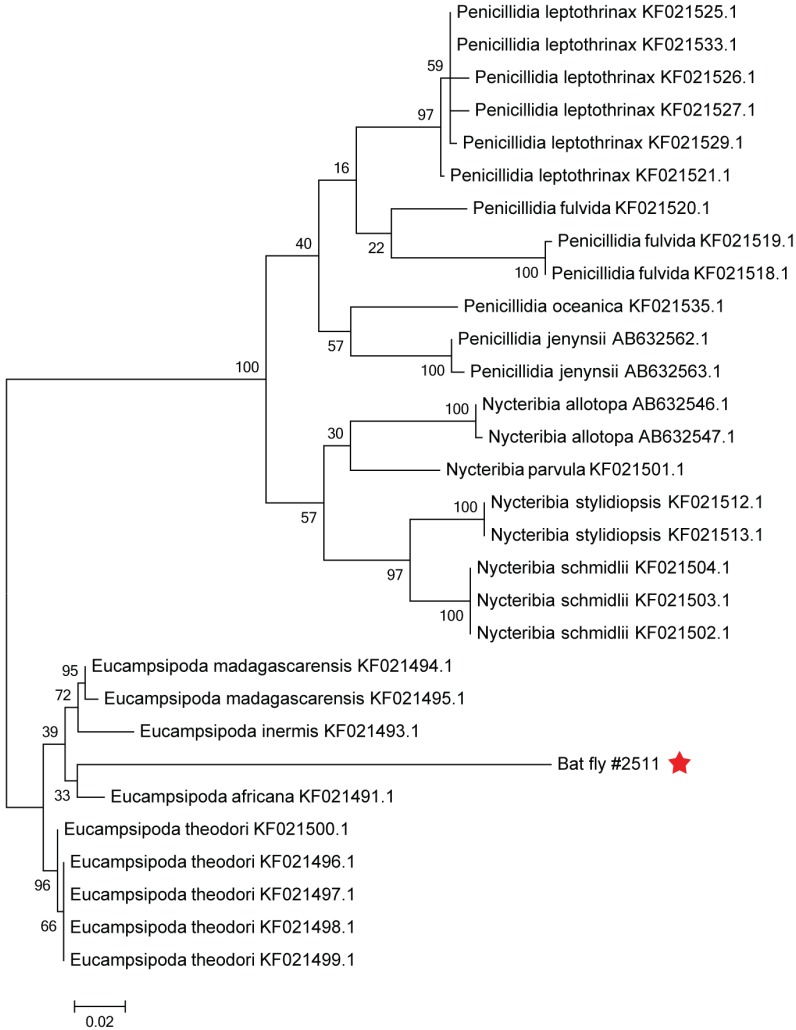
Phylogenetic tree (Maximum Likelihood) based on cytochrome c oxidase subunit I sequences. The COI partial sequence from the bat fly sequenced from this study is indicated by the red star.

From a total of 273 bat ectoparasite pools subjected to virus isolation by three blind passages, two yielded an agent that caused obvious cytopathic effects in the form of syncytia (giant cell) formation by three or four days post inoculation (d.p.i.) (Figure 2). The parasite pool that yielded MAHLV isolate 2511 was collected from an apparently healthy adult female *Rousettus aegyptiacus* bat captured at Mahune cave in May 2013. CPE in Vero cells were noted after two blind passages, and the supernatant collected on day five from passage four in a 75 cm^2^ flask containing infected Vero cells yielded 1 × 10^6.25^ TCID_50_/mL of the unknown virus. The second parasite pool that yielded MAHLV isolate 06-24 was collected from an apparently healthy juvenile male *Rousettus aegyptiacus* bat captured at Mahune cave in June 2013. CPE in Vero cells were noted after three blind passages, and the supernatant collected on day five from passage five in a 75 cm^2^ flask containing infected Vero cells yielded 1 × 10^6^ TCID_50_/mL of the virus. These supernatants, passage four of 2511 and passage five of 06-24, were used for subsequent identification by TEM and NGS. The ectoparasites from which the viruses were isolated were morphologically identified as bat flies, *Eucampsipoda africana* Theodor (*Diptera: Nycteribiidae*) (Figure 3) [44]. Sequencing of the cytochrome c oxidase subunit I gene (COI) and alignment to sequences available on Genbank, followed by phylogenetic analysis (Figure 4) confirms that the bat flies in this study are closest related to *Eucampsipoda spp.* identified before.

### 3.2. Identification as an Orthoreovirus by TEM and SISPA-NGS

**Figure 5:**
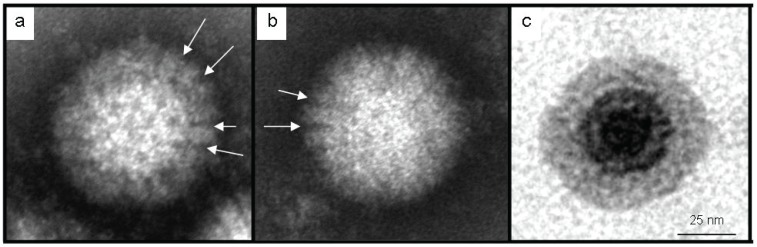
Virion ultrastructure of MAHLV isolate 06-24. (**a**) Negatively-stained particle with two distinct layers; (**b**) icosahedral negatively-stained particle; (**c**) resin-embedded, sectioned viral particle in cytoplasm of infected Vero E6 cell. White arrows indicate some of the characteristic spaces between the finger-like, capsomeric projections surrounding the solvent channels through the outer capsid layer.

Initial screening of negatively-stained culture supernatants revealed the presence of rounded icosahedrons lacking envelopes, which resembled non-Rotavirus-like virions of the *Reoviridae* (Figure 5). Two-layered capsids with an outer diameter of 70–75 nm (n = 30) and an inner core of 42–45 nm, possessed clearly defined solvent channels radiating outwards through the clustered capsomers of the outer capsid layer (Figure 5). Although the dimensions of the negatively-stained, inner capsid layer were comparable to those recorded after processing for ultramicrotomy, the outer layer was slightly larger, occasionally measuring up to 81 nm in diameter.

**Figure 6:**
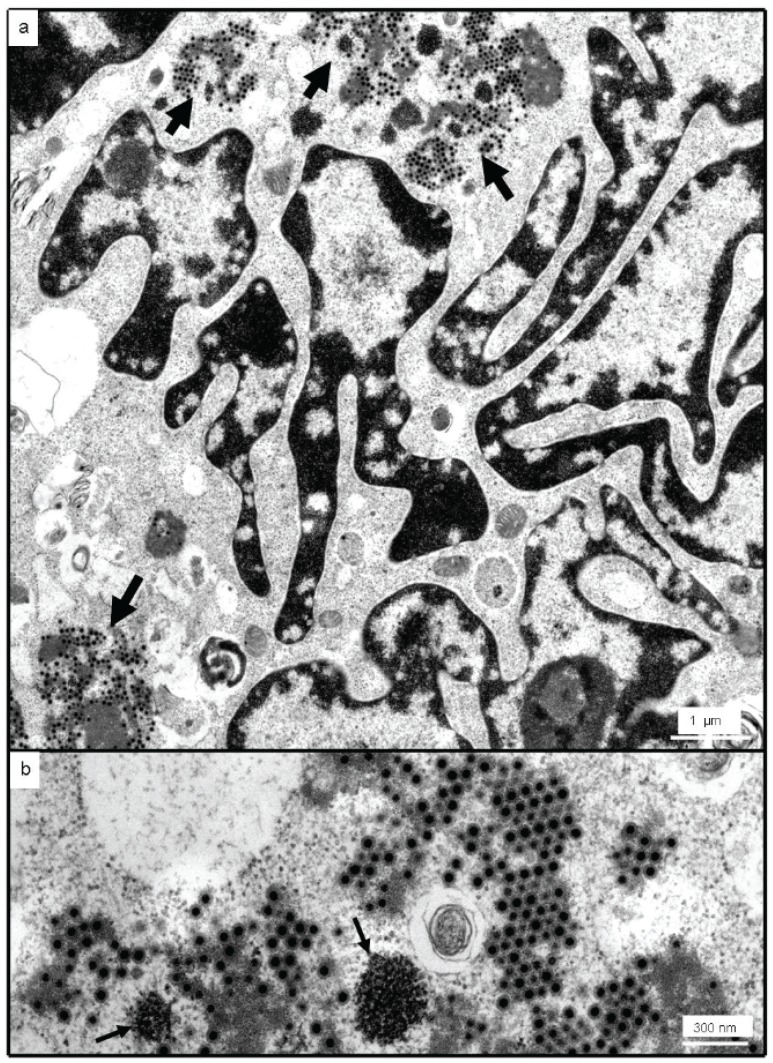
Vero E6 cells infected with MAHLV isolate 06-24. (**a**) multinucleate cells with lobed nuclei and scattered cytoplasmic inclusion bodies (arrows); (**b**) para-crystalline arrays of bi-layered virions developing from amorphous, grey inclusion bodies which were consistently associated with osmiophilic deposits resembling clusters of large ribosomes (arrows).

Evident in ultrathin sections of infected Vero E6 cells were multinucleate cells with extensive nuclear lobing, and cytoplasmic inclusion bodies associated with developing, double-shelled virus particles (Figure 6). TEM observations therefore suggested that the isolated virus belonged to the *Reoviridae*, sub-family *Spinareovirinae*.

An unbiased next-generation sequencing approach using SISPA amplification confirmed the presence of a novel Orthoreovirus. Initial sequencing results of both isolates yielded enough sequence coverage to identify all 10 segments. A polyetheleneglycol (PEG) precipitated preparation of isolate 06-24 yielded the most viral specific reads and formed 11 contigs aligning to Othoreoviruses using BLASTn and BLASTx. Both the 5' and 3' ends were missing for all the segments, so to obtain complete genomes for each isolate, rRNA depletion and a combination of SISPA and rapid amplification of cDNA ends (SISPA-RACE) was done. Read numbers were also increased by running samples on an Illumina Nextseq 500. Both an increase in the percentage of viral reads aligning to the genome segments and an increase in coverage of the ends were observed. Presence of MAHLV in the original homogenates from which the isolates were obtained was confirmed by SISPA amplification and NGS directly from the homogenates. Expectedly, only a low number of reads from both homogenates mapped to the MAHLV sequence due to the high amount of host sequence obscuring viral specific sequences combined with likely low viral load in the homogenates and the relatively low sensitivity of the SISPA method.

### 3.3. Genome Analysis and Phylogeny Reveals a Putative New African Orthoreovirus Species

**Figure 7:**
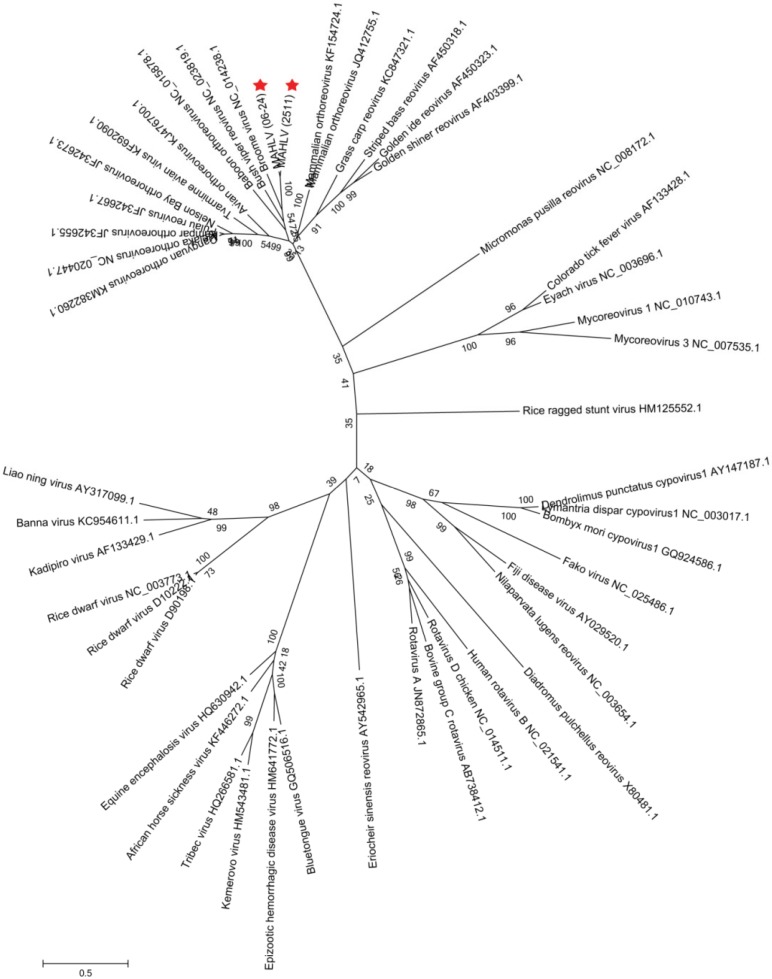
Molecular phylogenetic analysis by Maximum Likelihood method showing representative viruses from the *Reoviridae* family using the Lambda A (L1 segment) nucleic acid sequences. Genera to which the viruses belong are given in Table 1. The two MAHLV isolates are indicated by red stars and groups with other orthoreoviruses.

**Figure 8:**
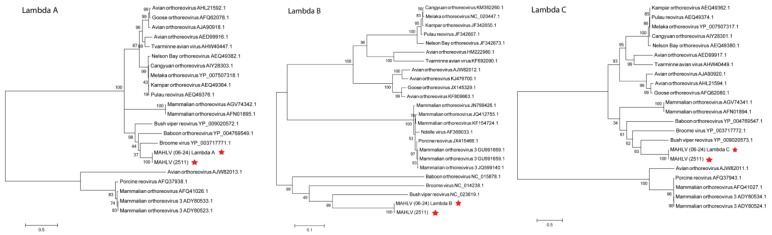
Molecular phylogenetic analysis by Maximum Likelihood method showing the placement of MAHLV within the *Orthoreovirus* genus using amino acid sequences of the ORFs in the three large segments, L1, 3 and 2 coding for proteins Lambda A, B and C.

**Figure 9:**
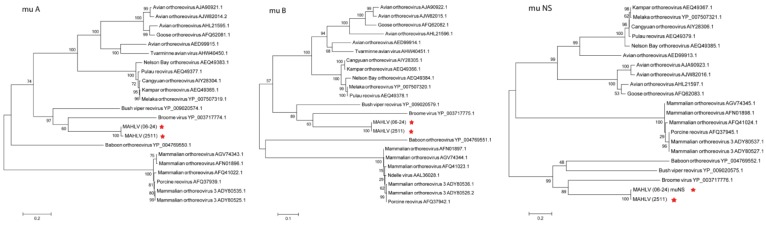
Molecular phylogenetic analysis by Maximum Likelihood method showing the placement of MAHLV within the *Orthoreovirus* genus using amino acid sequences of the ORFs in the three medium segments, M1, 2 and 3 coding for proteins mu A, B and NS.

**Figure 10:**
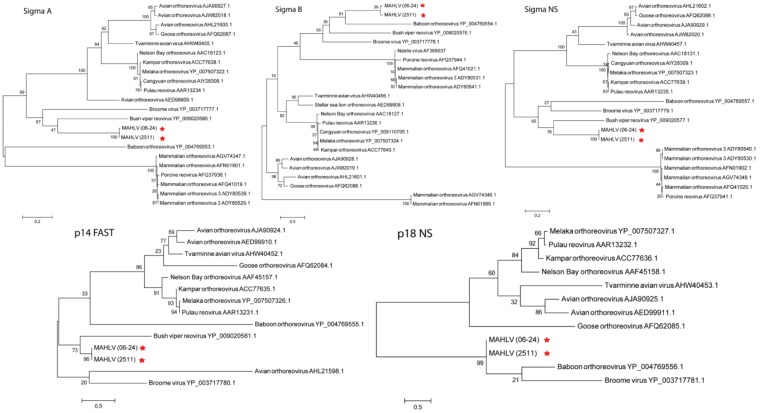
Molecular phylogenetic analysis by Maximum Likelihood method showing the placement of MAHLV within the *Orthoreovirus* genus using amino acid sequences of the five ORFs in the four small segments, S1, 2, 3 and 4 coding for proteins Sigma A, B, NS, and p14 FAST and p18 NS.

A Maximum Likelihood tree, constructed with nucleic acid sequence data for the RNA-dependent RNA polymerase (RdRp) encoding segments of representative viruses from the different genera within *Reoviridae* (Figure 7) shows the placement of both isolates amongst other orthoreoviruses in the family. Maximum Likelihood trees were prepared using the deduced amino acid sequences from the open reading frames (ORF’s) of all the virus’ segments and those of other viruses in the *Orthoreovirus* genus (Figure 8, Figure 9 and Figure 10). A distinct clade is formed by MAHLV, Bush viper reovirus, Baboon orthoreovirus and Broome virus within the genus. The above-mentioned clade is visibly distinct from others composed of bat-associated viruses; the Nelson Bay orthoreovirus and bat-derived mammalian orthoreoviruses. The closest relative of MAHLV, based on sequence homology of a conserved core protein, is Bush viper reovirus (Lambda B nucleic acid identity—63.7%; RdRp amino acid identity—66.3%) while the closest bat-associated virus is Broome virus (LambdaB nucleic acid identity—60.7%; RdRp amino acid identity—58.0%) (Table 2). Homology of the divergent major outer capsid protein of MAHLV to known orthoreoviruses is much lower: Sigma B nucleic acid identity—28.7%–41.9%; amino acid identity—5.6%–24.3% (Table 3).

The genome segments of MAHLV were named according to the nucleotide length, which is consistent with the nomenclature of other orthoreoviruses [26]. A summary of the MAHLV genome is given in Table 4. The total genome size is 23,200 nucleotides and predicted to encode eleven proteins, seven of which are structural. All ten genome segments of MAHLV contain an identical 3' terminal sequence, UCAUC-3', which is conserved between all known species of *Orthoreovirus*, and an identical 5' terminal sequence, 5'-GGUCA which is unique to MAHLV. Non-coding regions (NCRs) are present at both ends of the genome segments, with the 5' NCRs being shorter in nucleotide length than 3' NCRs. The nucleotide sequences of the two isolates of MAHLV, 2511 and 06-24, are not identical. Nucleotide homology of the RdRp encoding segment between the two isolates is 93.5% (99.8% deduced amino acid sequence), and 80.4% (89.0% deduced amino acid sequence) for the Sigma B encoding segment.

Putative protein functions were determined by BLASTx similarity searches to sequences available on Genbank, revealing putative functions known for other orthoreoviruses. The segments L2, L3, M1, M3, S1, S2 and S3 each contain a single start *AUG* codon in close proximity to the 5' end. The L1 segment of MAHLV contains two *AUG* start codons in close proximity to the 5' end, at positions 14 and 19. The first start codon (position 14) complies with Kozak’s rule (**A**CT*AUG***G**) and initiates an open reading frame 3885 nucleotides in length, putatively coding for Lambda A protein which is a core structural protein with NTPase, helicase and dsRNA binding ability. The second start codon (position 19) does not comply with Kozak’s rule (TGG*AUG***G**); it initiates a 402 nucleotide open reading frame but the deduced amino acid sequence does not match any known viral protein of note on Genbank. The M2 segment contains two *AUG* codons at positions 29 and 169, both complying with Kozak’s rule (**A**CG*AUG***G** and **A**GC*AUG***G**). The open reading frame initiating at position 29 is 2031 nucleotides in length and putatively encodes for an outer capsid protein involved in membrane penetration during infection (muB). The second *AUG* initiates a 492 nucleotide open reading frame but the deduced amino acid sequence does not match any viral protein of note on Genbank. The deduced Sigma A protein of MAHLV (segment S1) contains the fusogenic orthoreovirus-wide conserved arginine amino acid at position 273. The S4 segment is bicistronic and encodes for a 14 kDa protein similar to p14 membrane fusion protein of Bush viper reovirus and a non-overlapping 18 kDa protein similar to p16 non-structural protein of Baboon orthoreovirus without a known function (Table 4). The isoelectric point of MAHLV p18 is acidic (5.02), similar to that of p16 of Broome virus and Baboon orthoreovirus and contrary to that of other orthoreoviruses. The first ten amino acids in the putative 14 kDa protein of MAHLV are identical to the first ten amino acids in the p13 fusion protein of Broome virus and represent the myristoylation consensus sequence required for fusion activity of the protein. The S4 segment does not encode a cell attachment protein, an observation also characteristic of Broome virus and Baboon orthoreovirus.

### 3.4. Virus Growth in Mammalian and Insect Cells in Vitro

MAHLV replicated efficiently in Vero cell culture, with the inoculum containing a high dose of virus (10^5^ TCID_50_/mL) leading to rapid monolayer destruction after inoculation, with a peak in virus RNA (measured by real-time RT-PCR) by day 7, followed by a decrease on day 13. The inoculums containing a lower virus dose (10^4^ and 10^3^ TCID_50_/mL) resulted in a peak of RNA detection on day 13. All three above-mentioned inoculum doses yielded detectable virus RNA by day 4 after inoculation. The inoculum containing 10^1^ TCID_50_/mL virus did not generate detectable viral RNA until day 7, and was still showing an upward trend on day 13 (last sampling day). The virus did not replicate in the insect cells (C6-36) up to day 13, but adaptation to these cells through serial passaging was not attempted.

## 4. Discussion

The role of bats in harbouring pathogens of public health and veterinary importance is becoming an increasingly popular topic of research within the field of emerging and zoonotic diseases. The most notable viruses in which natural transmission from bats have been implicated include filoviruses, coronaviruses, paramyxoviruses, herpesviruses, lyssaviruses and bunyaviruses. The implication of bats in transmission or maintenance of some of these viruses is very circumstantial and often based only on serological evidence. More convincing evidence for others is based on detection of viral nucleic acid and isolation of live virus, although this does not conclusively prove that a vertebrate host is a reservoir. Finding pathogens in bats leads to the questions of how they are transmitted between bats, and from bats to incidental hosts such as humans. One possible transmission mechanism could be bat-associated hematophagous arthropods, such as the bat flies, but migration of parasites between bats is not well understood and would require further entomological investigation to better understand [13].

Two isolates of a novel fusogenic orthoreovirus were cultured and we determined their full genome sequences, which were compared to currently known viruses in the genus. The two isolates are not identical but similar enough to suggest that they are merely two isolates of the same virus. This suggests that there are multiple variants of the virus present in the host population. Members of a species within the *Orthoreovirus* genus are usually identified by a number of characteristics: amino acid and nucleotide sequence identity, organization of the polycistronic genome segment and host species [19]. For conserved core proteins, an amino acid identity >85% for homologous proteins indicates that two viruses belong to the same species, while identity <65% indicates a possible new species. When comparing the amino acid sequence of more divergent outer capsid proteins, >55% identity indicates one species and <35% indicates different species. Nucleic acid sequence identity of homologous segments of >75% indicates the same species and <60% a new species. The nature of conserved genome segment termini sequences of orthoreoviruses is also useful for virus classification [45]. The divergence of MAHLV sequence from other known orthoreoviruses combined with a unique conserved 5’ genome segment end and a unique host species, suggests that this is a new virus species in this genus. Along with Broome virus and Baboon orthoreovirus, MAHLV is the third orthoreovirus that lacks an identified cell attachment protein in the S4 segment. This unique characteristic further strengthens the phylogenetic classification which places these viruses in a separate clade and suggests that entry of these viruses into cells is mediated differently than for other orthoreoviruses.

Taking the abovementioned criteria and the sequence characteristics of the novel virus described here into consideration, we propose that Mahlapitsi virus constitutes a new species within the *Orthoreovirus* genus. To our knowledge this is the first description of an orthoreovirus in Africa with an indirect link to bats. Considering the rich diversity of bat species found on the continent and increased scientific interest in this field, this is unlikely to be the only such virus to be isolated from bat ectoparasites in years to come. However, to our knowledge this is the first orthoreovirus to be isolated from an arthropod host, since all currently known viruses in this genus are associated with vertebrates. MAHLV did not replicate on C6-36 cells in this study but *Aedes albopictus*, from which the C6-36 cell line is derived, is classified in a completely different dipteran family, the *Culicidae*, likely pointing to a cell receptor incompatibility. Another possible explanation could be the temperature at which insect cells are cultured compared to mammalian cells, which might be incompatible with this virus.

The arthropod-borne nature of MAHLV transmission needs further investigation, especially to establish whether nycteribiid flies are only involved in mechanical or possibly biological transmission, and if the virus is even transmitted to bats. Various other genera in the *Reoviridae* family contain vector-borne viruses, including Banna virus (*Seadornavirus*), Colorado tick fever virus (*Coltivirus*) and Bluetongue virus (*Orbivirus*). Our isolation of MAHLV from arthropods might direct some attention to the possible role of insects in the transmission of currently known orthoreoviruses, or possibly the presence of other yet unknown viruses in various arthropods. We have no information on the geographical range of MAHLV, but the wide distribution of *Rousettus aegyptiacus* in Africa and the Middle East [46], and the strict host preference and specificity of bat flies [13,14,15], dictate that their ranges will overlap.

We have no data to suggest that MAHLV has any human health implication, but this warrants further investigation. The virus grows to high titers in Vero E6 cells which suggests that it may infect vertebrates, although growth in *in vitro* systems cannot be translated directly into replication in a vertebrate host. It is important to note, also, that this was after blind passage and cell culture adaptation (CPE noted after two–three blind passages). Any risk of human infection for now, however, is only likely in individuals who come into close contact with wild Egyptian fruit bats and their ectoparasites. Although highly host-dependent, the bat flies have been noted to leave their bat hosts and crawl on bat researchers (personal observation). Respiratory disease has been noted in humans infected with Melaka, Kampar and Nelson bay orthoreovirus, including limited human-to-human transmission [22,23,32,33]. Thus the potential for MAHLV to infect humans, and spread between humans, cannot be excluded until further investigation is done, especially considering that the virus grows very efficiently on a monkey-derived cell line.

## 5. Conclusions

In conclusion, we have identified a novel orthoreovirus which we propose should constitute a new species within the genus. Two virus strains were isolated from ectoparasitic bat flies collected from Egyptian fruit bats from a South African cave roost. This represents the first isolation of an orthoreovirus from arthropods and the first African virus in this genus with an indirect link to bats.

## Figures and Tables

**Table 1:** Genbank accession numbers of sequences used for phylogenetic analysis in this study.

Genbank Accession Number	Subfamily/ Family	Genus	Virus/Species (Isolate)	Gene Description (Encoding for Protein)	Nucleic Acid (NA) or Amino Acid (AA)
AF450323.1	*Spinareovirinae*	*Aquareovirus*	Golden ide reovirus	RNA dependent RNA polymerase (RdRp)	NA
AF403399.1	*Spinareovirinae*	*Aquareovirus*	Golden shiner reovirus	RdRp	NA
KC847321.1	*Spinareovirinae*	*Aquareovirus*	Grass carp reovirus (HeNan988)	RdRp	NA
AF450318.1	*Spinareovirinae*	*Aquareovirus*	Striped bass reovirus	RdRp	NA
AY542965.1	*Sedoreovirinae*	*Cardoreovirus*	Eriocheir sinensis reovirus	RdRp	NA
AF133428.1	*Spinareovirinae*	*Coltivirus*	Colorado tick fever virus	RdRp	NA
NC_003696.1	*Spinareovirinae*	*Coltivirus*	Eyach virus	RdRp	NA
GQ924586.1	*Spinareovirinae*	*Cypovirus*	Bombyx mori cypovirus1	RdRp	NA
AY147187.1	*Spinareovirinae*	*Cypovirus*	Dendrolimus punctatus cypovirus1	RdRp	NA
NC_003017.1	*Spinareovirinae*	*Cypovirus*	Lymantria dispar cypovirus1	RdRp	NA
NC_025486.1	*Spinareovirinae*	*Dinovernavirus*	Fako virus (CSW77)	RdRp	NA
AY029520.1	*Spinareovirinae*	*Fijivirus*	Fiji disease virus	RdRp	NA
NC_003654.1	*Spinareovirinae*	*Fijivirus*	Nilaparvata lugens reovirus	RdRp	NA
X80481.1	*Spinareovirinae*	*Idnoreovirus*	Diadromus pulchellus reovirus	RdRp	NA
NC_008172.1	*Sedoreovirinae*	*Mimoreovirus*	Micromonas pusilla reovirus	RdRp	NA
NC_010743.1	*Spinareovirinae*	*Mycoreovirus*	Mycoreovirus 1	RdRp	NA
NC_007535.1	*Spinareovirinae*	*Mycoreovirus*	Mycoreovirus 3	RdRp	NA
KF446272.1	*Sedoreovirinae*	*Orbivirus*	African horse sickness virus (RSArrah/08)	RdRp	NA
GQ506516.1	*Sedoreovirinae*	*Orbivirus*	Bluetongue virus (BT2-03)	RdRp	NA
HM641772.1	*Sedoreovirinae*	*Orbivirus*	Epizootic hemorrhagic disease virus (CC304-06)	RdRp	NA
HQ630942.1	*Sedoreovirinae*	*Orbivirus*	Equine encephalosis virus (Kyalami)	RdRp	NA
HM543481.1	*Sedoreovirinae*	*Orbivirus*	Kemerovo virus (EgAn 1169-61)	RdRp	NA
HQ266581.1	*Sedoreovirinae*	*Orbivirus*	Tribec virus	RdRp	NA
KJ476700.1	*Spinareovirinae*	*Orthoreovirus*	Avian orthoreovirus (GX/2010/1)	RdRp	NA
NC_015878.1	*Spinareovirinae*	*Orthoreovirus*	Baboon orthoreovirus	RdRp	NA
NC_014238.1	*Spinareovirinae*	*Orthoreovirus*	Broome virus	RdRp	NA
NC_023819.1	*Spinareovirinae*	*Orthoreovirus*	Bush viper reovirus	RdRp	NA
KM382260.1	*Spinareovirinae*	*Orthoreovirus*	Cangyuan orthoreovirus	RdRp	NA
JF342655.1	*Spinareovirinae*	*Orthoreovirus*	Kampar orthoreovirus	RdRp	NA
KF154724.1	*Spinareovirinae*	*Orthoreovirus*	Mammalian orthoreovirus (SI-MRV01)	RdRp	NA
JQ412755.1	*Spinareovirinae*	*Orthoreovirus*	Mammalian Orthoreovirus (T3/Bat/Germany/342/08)	RdRp	NA
NC_020447.1	*Spinareovirinae*	*Orthoreovirus*	Melaka orthoreovirus	RdRp	NA
JF342673.1	*Spinareovirinae*	*Orthoreovirus*	Nelson Bay orthoreovirus	RdRp	NA
JF342667.1	*Spinareovirinae*	*Orthoreovirus*	Pulau reovirus	RdRp	NA
KF692090.1	*Spinareovirinae*	*Orthoreovirus*	Tvarminne avian virus	RdRp	NA
HM125552.1	*Spinareovirinae*	*Oryzavirus*	Rice ragged stunt virus	RdRp	NA
D90198.1	*Spinareovirinae*	*Oryzavirus*	Rice dwarf virus	RdRp	NA
NC_003773.1	*Spinareovirinae*	*Oryzavirus*	Rice dwarf virus	RdRp	NA
D10222.1	*Spinareovirinae*	*Oryzavirus*	Rice dwarf virus	RdRp	NA
AB738412.1	*Sedoreovirinae*	*Rotavirus*	Bovine group C rotavirus (Toyama)	RdRp	NA
NC_021541.1	*Sedoreovirinae*	*Rotavirus*	Human rotavirus B (Bang373)	RdRp	NA
JN872865.1	*Sedoreovirinae*	*Rotavirus*	Rotavirus A (RVA/Horse-wt/ARG/E4040/2008/G14P12)	RdRp	NA
NC_014511.1	*Sedoreovirinae*	*Rotavirus*	Rotavirus D chicken (05V0049/DEU/2005)	RdRp	NA
KC954611.1	*Sedoreovirinae*	*Seadornavirus*	Banna virus	RdRp	NA
AF133429.1	*Sedoreovirinae*	*Seadornavirus*	Kadipiro virus	RdRp	NA
AY317099.1	*Sedoreovirinae*	*Seadornavirus*	Liao ning virus (LNSV-NE97-31)	RdRp	NA
AJA90918.1 KJ476700.1 AJA90920.1 AJA90921.1 AJA90922.1 AJA90923.1 AJA90927.1 AJA90928.1 AJA90929.1 AJA90924.1 AJA90925.1	*Spinareovirinae*	*Orthoreovirus*	Avian orthoreovirus (GX/2010/1)	Virus structural and non-structural proteins	AA
YP_004769549.1 NC_015878.1 YP_004769547.1 YP_004769550.1 YP_004769551.1 YP_004769552.1 YP_004769553.1 YP_004769554.1 YP_004769557.1 YP_004769555.1 YP_004769556.1	*Spinareovirinae*	*Orthoreovirus*	Baboon orthoreovirus	Virus structural and non-structural proteins	AA
YP_003717771.1 NC_014238.1 YP_003717772.1 YP_003717774.1 YP_003717775.1 YP_003717776.1 YP_003717777.1 YP_003717778.1 YP_003717779.1 YP_003717780.1 YP_003717781.1	*Spinareovirinae*	*Orthoreovirus*	Broome virus	Virus structural and non-structural proteins	AA
YP_009020572.1 NC_023819.1 YP_009020573.1 YP_009020574.1 YP_009020579.1 YP_009020575.1 YP_009020580.1 YP_009020576.1 YP_009020577.1 YP_009020581.1	*Spinareovirinae*	*Orthoreovirus*	Bush viper reovirus	Virus structural and non-structural proteins	AA*
AIY28303.1 KM382260.1 AIY28301.1 AIY28304.1 AIY28305.1 AIY28306.1 AIY28308.1 YP_009110705.1 AIY28309.1	*Spinareovirinae*	*Orthoreovirus*	Cangyuan orthoreovirus	Virus structural and non-structural proteins	AA*
AEQ49364.1 JF342655.1 AEQ49362.1 AEQ49365.1 AEQ49366.1 AEQ49367.1 ACC77638.1 ACC77640.1 ACC77639.1 ACC77635.1 ACC77636.1	*Spinareovirinae*	*Orthoreovirus*	Kampar orthoreovirus	Virus structural and non-structural proteins	AA
AGV74342.1 KF154724.1 AGV74341.1 AGV74343.1 AGV74344.1 AGV74345.1 AGV74347.1 AGV74346.1 AGV74348.1	*Spinareovirinae*	*Orthoreovirus*	Mammalian orthoreovirus (SI-MRV01)	Virus structural and non-structural proteins	AA*
AFN01895.1 JQ412755.1 AFN01894.1 AFN01896.1 AFN01897.1 AFN01898.1 AFN01901.1 AFN01899.1 AFN01902.1	*Spinareovirinae*	*Orthoreovirus*	Mammalian Orthoreovirus (T3/Bat/Germany/342/08)	Virus structural and non-structural proteins	AA*
YP_007507318.1 NC_020447.1 YP_007507317.1 YP_007507319.1 YP_007507320.1 YP_007507321.1 YP_007507322.1 YP_007507324.1 YP_007507323.1 YP_007507326.1 YP_007507327.1	*Spinareovirinae*	*Orthoreovirus*	Melaka orthoreovirus	Virus structural and non-structural proteins	AA
AEQ49382.1 JF342673.1 AEQ49380.1 AEQ49383.1 AEQ49384.1 AEQ49385.1 AAC18123.1 AAC18127.1 AAC18131.1 AAF45157.1 AAF45158.1	*Spinareovirinae*	*Orthoreovirus*	Nelson Bay orthoreovirus	Virus structural and non-structural proteins	AA
AEQ49376.1 JF342667.1 AEQ49374.1 AEQ49377.1 AEQ49378.1 AEQ49379.1 AAR13234.1 AAR13236.1 AAR13235.1 AAR13231.1 AAR13232.1	*Spinareovirinae*	*Orthoreovirus*	Pulau reovirus	Virus structural and non-structural proteins	AA
AHW40447.1 KF692090.1 AHW40449.1 AHW40450.1 AHW40451.1 AHW40455.1 AHW40456.1 AHW40457.1 AHW40452.1 AHW40453.1	*Spinareovirinae*	*Orthoreovirus*	Tvarminne avian virus	Virus structural and non-structural proteins	AA*
AED99916.1 HM222980.1 AED99917.1 AED99915.1 AED99914.1 AED99913.1 AED99909.1 AED99908.1 AED99910.1 AED99911.1	*Spinareovirinae*	*Orthoreovirus*	Stellar sea lion orthoreovirus	Virus structural and non-structural proteins	AA*
AHL21592.1 KF809663.1 AHL21594.1 AHL21595.1 AHL21596.1 AHL21597.1 AHL21600.1 AHL21601.1 AHL21602.1 AHL21598.1	*Spinareovirinae*	*Orthoreovirus*	Avian orthoreovirus (D20/99)	Virus structural and non-structural proteins	AA*
AJW82013.1 AJW82012.1 AJW82011.1 AJW82014.2 AJW82015.1 AJW82016.1 AJW82018.1 AJW82019.1 AJW82020.1	*Spinareovirinae*	*Orthoreovirus*	Avian orthoreovirus (PA/Turkey/22342/13)	Virus structural and non-structural proteins	AA*
AFQ62078.1 JX145329.1 AFQ62080.1 AFQ62081.1 AFQ62082.1 AFQ62083.1 AFQ62087.1 AFQ62088.1 AFQ62089.1 AFQ62084.1 AFQ62085.1	*Spinareovirinae*	*Orthoreovirus*	Goose orthoreovirus (03G)	Virus structural and non-structural proteins	AA
ADY80533.1 GU991669.1 ADY80534.1 ADY80535.1 ADY80536.1 ADY80537.1 ADY80539.1 ADY80541.1 ADY80540.1	*Spinareovirinae*	*Orthoreovirus*	Mammalian orthoreovirus (3jin-1)	Virus structural and non-structural proteins	AA*
ADY80523.1 GU991659.1 ADY80524.1 ADY80525.1 ADY80526.2 ADY80527.1 ADY80529.1 ADY80531.1 ADY80530.1	*Spinareovirinae*	*Orthoreovirus*	Mammalian orthoreovirus 3 (R124)	Virus structural and non-structural proteins	AA*
JQ599140.1	*Spinareovirinae*	*Orthoreovirus*	Mammalian orthoreovirus 3 (T3v1)	Virus structural and non-structural proteins	AA*
AFQ41026.1 JN799426.1 AFQ41027.1 AFQ41022.1 AFQ41023.1 AFQ41024.1 AFQ41019.1 AFQ41021.1 AFQ41020.1	*Spinareovirinae*	*Orthoreovirus*	Mammalian orthoreovirus (729)	Virus structural and non-structural proteins	AA*
AF368033.1 AAL36028.1 AF368037	*Spinareovirinae*	*Orthoreovirus*	Ndelle virus	Virus structural and non-structural proteins	AA*
AFQ37938.1 JX415466.1 AFQ37943.1 AFQ37939.1 AFQ37942.1 AFQ37945.1 AFQ37936.1 AFQ37944.1 AFQ37941.1	*Spinareovirinae*	*Orthoreovirus*	Porcine reovirus (SHR-A)	Virus structural and non-structural proteins	AA*
KF021491.1	*Nycteribiidae*	*Eucampsipoda*	*africana* (isolate N4)	COI	NA
KF021493.1	*Nycteribiidae*	*Eucampsipoda*	*inermis* (isolate N8)	COI	NA
KF021494.1	*Nycteribiidae*	*Eucampsipoda*	*madagascarensis* (isolate J43)	COI	NA
KF021495.1	*Nycteribiidae*	*Eucampsipoda*	*madagascarensis* (isolate J48)	COI	NA
KF021500.1	*Nycteribiidae*	*Eucampsipoda*	*theodori* (isolate 8B)	COI	NA
KF021496.1	*Nycteribiidae*	*Eucampsipoda*	*theodori* (isolate 28DM)	COI	NA
KF021497.1	*Nycteribiidae*	*Eucampsipoda*	*theodori* (isolate 29F)	COI	NA
KF021498.1	*Nycteribiidae*	*Eucampsipoda*	*theodori* (isolate 30DM)	COI	NA
KF021499.1	*Nycteribiidae*	*Eucampsipoda*	*theodori* (isolate 31DM)	COI	NA
AB632546.1	*Nycteribiidae*	*Nycteribia*	*allotopa* (isolate NyAl10)	COI	NA
AB632547.1	*Nycteribiidae*	*Nycteribia*	*allotopa* (isolate NyAl11)	COI	NA
KF021501.1	*Nycteribiidae*	*Nycteribia*	*parvula* (isolate N16)	COI	NA
KF021503.1	*Nycteribiidae*	*Nycteribia*	*schmidlii* (isolate N2)	COI	NA
KF021504.1	*Nycteribiidae*	*Nycteribia*	*schmidlii* (isolate N3)	COI	NA
KF021502.1	*Nycteribiidae*	*Nycteribia*	*schmidlii* (isolate N21)	COI	NA
KF021512.1	*Nycteribiidae*	*Nycteribia*	*stylidiopsis* (isolate J32)	COI	NA
KF021513.1	*Nycteribiidae*	*Nycteribia*	*stylidiopsis* (isolate J33)	COI	NA
KF021519.1	*Nycteribiidae*	*Penicillidia*	*fulvida* (isolate GR21)	COI	NA
KF021518.1	*Nycteribiidae*	*Penicillidia*	*fulvida* (isolate J68)	COI	NA
KF021520.1	*Nycteribiidae*	*Penicillidia*	*fulvida* (isolate N20)	COI	NA
AB632562.1	*Nycteribiidae*	*Penicillidia*	*jenynsii* (isolate PeJe6)	COI	NA
AB632563.1	*Nycteribiidae*	*Penicillidia*	*jenynsii* (isolate PeJe7)	COI	NA
KF021525.1	*Nycteribiidae*	*Penicillidia*	*leptothrinax* (isolate 35A)	COI	NA
KF021526.1	*Nycteribiidae*	*Penicillidia*	*leptothrinax* (isolate 35B)	COI	NA
KF021527.1	*Nycteribiidae*	*Penicillidia*	*leptothrinax* (isolate 36A)	COI	NA
KF021521.1	*Nycteribiidae*	*Penicillidia*	*leptothrinax* (isolate GR7)	COI	NA
KF021529.1	*Nycteribiidae*	*Penicillidia*	*leptothrinax* (isolate J34)	COI	NA
KF021533.1	*Nycteribiidae*	*Penicillidia*	*leptothrinax* (isolate J66)	COI	NA
KF021535.1	*Nycteribiidae*	*Penicillidia*	*oceanica* (isolate N17)	COI	NA

* The star denotes viruses for which amino acid sequences were not available on Genbank for the full range of viral structural and non-structural proteins.

**Table 2:** Identity values (%) of the RNA dependent RNA polymerase encoding segments and deduced amino acid sequences of MAHLV and other orthoreoviruses. Nucleic acid sequences are in the bottom left triangle and in bold text, and deduced amino acid sequences are in the top right triangle in italics. The grey shaded areas indicate the identity values of the closest related virus(es) to MAHLV.

Virus (Isolate Number)	1	2	3	4	5	6	7	8	9	10	11	12	13	14	15	16	17	18	19	20	21	22	23	24
**1.** **MAHLV (06-24)**		*99.8*	*56.6*	*53.6*	*58.0*	*66.3*	*55.6*	*55.5*	*54.6*	*54.7*	*55.6*	*55.2*	*55.6*	*57.7*	*57.5*	*57.2*	*56.6*	*56.6*	*54.5*	*54.5*	*54.4*	*54.5*	*54.4*	*54.4*
**2.** **MAHLV (2511)**	**93.5**		*56.6*	*53.6*	*58.0*	*66.3*	*55.6*	*55.5*	*54.6*	*54.7*	*55.6*	*55.2*	*55.6*	*57.7*	*57.5*	*57.2*	*56.5*	*56.6*	*54.5*	*54.5*	*54.4*	*54.5*	*54.4*	*54.4*
**3.** **Avian orthoreovirus (GX/2010/1)**	**57.0**	**56.5**		*50.0*	*50.9*	*55.4*	*70.6*	*70.5*	*54.8*	*54.9*	*70.5*	*70.2*	*70.5*	*71.3*	*72.1*	*91.4*	*95.2*	*91.2*	*54.8*	*54.8*	*54.8*	*54.9*	*54.8*	*54.8*
**4.** **Baboon orthoreovirus**	**58.0**	**58.1**	**54.4**		*50.5*	*52.8*	*49.6*	*49.5*	*47.5*	*47.5*	*49.4*	*49.8*	*49.3*	*50.0*	*50.4*	*50.3*	*50.1*	*50.0*	*47.4*	*47.4*	*47.4*	*47.6*	*47.6*	*47.4*
**5.** **Broome virus**	**60.2**	**60.7**	**55.0**	**58.9**		*56.0*	*50.5*	*50.4*	*50.7*	*50.7*	*50.6*	*50.0*	*50.4*	*51.4*	*50.4*	*51.4*	*51.5*	*51.2*	*50.7*	*50.7*	*50.6*	*50.4*	*50.4*	*50.8*
**6.** **Bush viper reovirus**	**63.7**	**63.3**	**55.9**	**56.5**	**58.2**		*57.0*	*56.9*	*51.8*	*51.8*	*56.9*	*56.8*	*56.6*	*56.8*	*56.1*	*55.5*	*54.8*	*55.3*	*51.9*	*51.9*	*51.9*	*51.7*	*51.5*	*51.8*
**7.** **Cangyuan orthoreovirus**	**56.6**	**56.6**	**63.8**	**52.9**	**53.4**	**56.8**		*99.4*	*53.3*	*53.5*	*99.4*	*95.9*	*98.8*	*73.3*	*72.5*	*71.3*	*69.8*	*71.3*	*53.4*	*53.3*	*53.3*	*53.5*	*53.3*	*53.4*
**8.** **Kampar orthoreovirus**	**56.5**	**56.6**	**63.7**	**52.6**	**53.3**	**56.6**	**98.0**		*53.2*	*53.3*	*99.2*	*95.6*	*98.8*	*73.3*	*72.3*	*71.3*	*69.7*	*71.2*	*53.2*	*53.2*	*53.2*	*53.3*	*53.2*	*53.2*
**9.** **Mammalian orthoreovirus (SI-MRV01)**	**56.8**	**57.4**	**54.6**	**53.7**	**55.2**	**55.6**	**53.0**	**52.8**		*99.4*	*53.3*	*52.4*	*53.1*	*53.4*	*53.1*	*54.4*	*54.4*	*53.9*	*98.9*	*98.8*	*98.6*	*98.7*	*98.3*	*99.1*
**10.** **Mammalian Orthoreovirus (T3/Bat/Germany/342/08)**	**57.2**	**57.6**	**54.7**	**54.0**	**55.2**	**55.5**	**52.8**	**52.7**	**98.3**		*53.5*	*52.6*	*53.2*	*53.6*	*53.2*	*54.5*	*54.6*	*54.0*	*98.6*	*98.5*	*98.3*	*98.4*	*98.1*	*98.8*
**11.** **Melaka orthoreovirus**	**56.5**	**56.4**	**63.6**	**52.8**	**53.5**	**56.5**	**97.9**	**98.6**	**52.6**	**52.5**		*95.6*	*98.6*	*73.2*	*72.1*	*71.2*	*69.7*	*71.1*	*53.4*	*53.3*	*53.3*	*53.3*	*53.3*	*53.4*
**12.** **Nelson Bay orthoreovirus**	**56.6**	**56.4**	**63.9**	**52.6**	**53.6**	**56.8**	**83.8**	**83.6**	**53.3**	**53.4**	**83.8**		*96.0*	*72.5*	*71.6*	*71.0*	*70.0*	*70.9*	*52.5*	*52.4*	*52.4*	*52.6*	*52.5*	*52.5*
**13.** **Pulau reovirus**	**55.9**	**55.9**	**63.6**	**52.5**	**53.2**	**56.6**	**94.0**	**94.6**	**53.2**	**53.0**	**94.6**	**83.6**		*73.2*	*72.1*	*71.2*	*69.7*	*71.1*	*53.2*	*53.1*	*53.1*	*53.2*	*53.1*	*53.2*
**14.** **Tvarminne avian virus**	**56.3**	**56.4**	**63.7**	**53.9**	**53.6**	**56.9**	**65.3**	**65.3**	**54.6**	**54.6**	**65.3**	**64.1**	**65.7**		*82.0*	*71.4*	*71.2*	*71.1*	*53.4*	*53.4*	*53.2*	*53.5*	*53.2*	*53.4*
**15.** **Avian orthoreovirus**	**57.5**	**57.4**	**64.2**	**52.7**	**53.6**	**56.1**	**65.3**	**65.3**	**54.9**	**54.8**	**65.4**	**64.6**	**65.1**	**70.2**		*72.1*	*72.1*	*71.5*	*53.2*	*53.2*	*53.1*	*53.2*	*52.7*	*53.2*
**16.** **Avian orthoreovirus (D20/99)**	**55.9**	**56.2**	**75.5**	**52.6**	**53.1**	**54.9**	**64.8**	**64.7**	**54.5**	**54.5**	**64.6**	**64.5**	**64.3**	**64.7**	**65.3**		*91.2*	*97.3*	*54.4*	*54.4*	*54.3*	*54.6*	*54.4*	*54.4*
**17.** **Avian orthoreovirus (PA/Turkey/22342/13)**	**56.8**	**56.8**	**83.6**	**52.6**	**54.5**	**54.8**	**63.1**	**63.0**	**54.3**	**53.9**	**63.1**	**63.8**	**63.3**	**63.6**	**64.7**	**75.4**		*90.7*	*54.5*	*54.5*	*54.4*	*54.6*	*54.4*	*54.5*
**18.** **Goose orthoreovirus (03G)**	**55.9**	**56.3**	**75.7**	**53.2**	**53.3**	**54.7**	**64.3**	**64.3**	**53.9**	**54.0**	**64.3**	**64.7**	**64.2**	**64.1**	**65.1**	**91.8**	**75.9**		*53.9*	*53.9*	*53.8*	*54.1*	*54.0*	*53.9*
**19.** **Mammalian orthoreovirus (3jin-1)**	**56.6**	**56.9**	**54.7**	**53.6**	**55.6**	**55.4**	**54.0**	**53.8**	**89.8**	**89.8**	**53.7**	**53.2**	**54.0**	**54.8**	**54.5**	**54.0**	**54.6**	**53.8**		*99.8*	*99.7*	*98.3*	*97.8*	*99.7*
**20.** **Mammalian orthoreovirus 3 (R124)**	**56.6**	**56.9**	**54.7**	**53.6**	**55.6**	**55.4**	**54.0**	**53.8**	**89.8**	**89.8**	**53.7**	**53.2**	**54.0**	**54.8**	**54.5**	**54.1**	**54.6**	**53.8**	**99.9**		*99.7*	*98.2*	*97.7*	*99.7*
**21.** **Mammalian orthoreovirus 3 (T3v1)**	**56.5**	**56.8**	**54.7**	**53.6**	**55.6**	**55.4**	**53.9**	**53.7**	**89.8**	**89.8**	**53.7**	**53.2**	**53.9**	**54.7**	**54.4**	**54.0**	**54.6**	**53.8**	**99.9**	**99.8**		*98.1*	*97.5*	*99.5*
**22.** **Mammalian orthoreovirus (729)**	**56.9**	**57.2**	**54.9**	**53.6**	**55.6**	**55.4**	**53.7**	**53.5**	**91.6**	**91.9**	**53.3**	**53.4**	**53.5**	**54.4**	**54.5**	**54.3**	**54.5**	**53.7**	**90.1**	**90.1**	**90.0**		*97.7*	*98.6*
**23.** **Ndelle virus**	**57.0**	**57.0**	**54.9**	**53.1**	**54.9**	**55.2**	**53.9**	**53.6**	**89.8**	**90.2**	**53.7**	**53.0**	**53.5**	**54.6**	**54.5**	**54.2**	**54.3**	**54.0**	**89.4**	**89.4**	**89.4**	**90.4**		*98.0*
**24.** **Porcine reovirus (SHR-A)**	**56.3**	**56.8**	**54.7**	**53.6**	**55.8**	**55.3**	**54.3**	**54.0**	**90.7**	**90.8**	**54.0**	**53.3**	**54.2**	**54.9**	**54.5**	**54.0**	**54.5**	**53.7**	**98.2**	**98.1**	**98.1**	**91.8**	**90.6**	

**Table 3:** Identity values (%) of the Sigma B encoding segments and deduced amino acid sequences of MAHLV and other orthoreoviruses. Nucleic acid sequences are in the bottom left triangle and in bold text, and deduced amino acid sequences are in the top right triangle in italics. The grey shaded areas indicate the identity values of the closest related virus(es) to MAHLV.

Virus (Isolate)	1	2	3	4	5	6	7	8	9	10	11	12	13	14	15	16	17	18	19	20	21	22	23
**1.** **MAHLV (06-24)**		*89.0*	*21.4*	*24.0*	*24.3*	*19.0*	*17.8*	*17.8*	*8.3*	*8.3*	*17.8*	*17.5*	*18.1*	*19.3*	*17.5*	*21.1*	*21.4*	*19.3*	*7.7*	*8.0*	*7.7*	*5.6*	*6.2*
**2.** **MAHLV (2511)**	**80.4**		*21.7*	*24.3*	*23.1*	*20.2*	*17.5*	*17.5*	*5.6*	*5.9*	*17.5*	*17.8*	*17.5*	*18.7*	*18.4*	*21.7*	*21.1*	*19.6*	*5.6*	*5.9*	*8.6*	*5.0*	*7.1*
**3.** **Avian orthoreovirus (GX/2010/1)**	**39.8**	**39.9**		*15.1*	*20.2*	*22.6*	*34.4*	*35.0*	*8.9*	*8.9*	*34.7*	*35.0*	*33.8*	*37.1*	*38.3*	*61.7*	*78.6*	*70.0*	*9.2*	*9.2*	*5.9*	*8.0*	*7.1*
**4.** **Baboon orthoreovirus**	**41.7**	**41.7**	**35.8**		*19.3*	*17.2*	*17.5*	*16.9*	*7.1*	*7.1*	*17.2*	*16.9*	*16.0*	*17.2*	*17.5*	*16.3*	*14.8*	*15.7*	*6.5*	*6.8*	*7.7*	*7.1*	*6.8*
**5.** **Broome virus**	**41.9**	**41.5**	**39.0**	**38.5**		*20.2*	*18.1*	*17.8*	*8.9*	*8.9*	*18.1*	*17.5*	*18.7*	*19.6*	*19.6*	*17.2*	*18.4*	*19.0*	*7.7*	*7.7*	*6.2*	*7.4*	*8.3*
**6.** **Bush viper reovirus**	**38.7**	**39.3**	**39.5**	**37.3**	**35.8**		*18.4*	*18.1*	*3.9*	*3.9*	*18.4*	*18.1*	*17.5*	*17.8*	*18.1*	*22.6*	*21.7*	*22.0*	*3.6*	*3.6*	*5.3*	*5.0*	*6.2*
**7.** **Cangyuan orthoreovirus**	**38.0**	**38.6**	**45.4**	**36.5**	**34.0**	**36.5**		*97.0*	*8.9*	*8.9*	*98.8*	*93.5*	*93.8*	*40.1*	*40.4*	*31.8*	*34.4*	*32.9*	*8.3*	*8.3*	*6.5*	*7.1*	*6.5*
**8.** **Kampar orthoreovirus**	**37.8**	**38.7**	**45.4**	**37.0**	**33.9**	**36.0**	**92.5**		*9.5*	*9.5*	*97.6*	*91.7*	*92.0*	*40.1*	*40.4*	*32.3*	*35.0*	*34.1*	*8.6*	*8.6*	*6.5*	*7.7*	*6.8*
**9.** **Mammalian orthoreovirus (SI-MRV01)**	**29.4**	**30.2**	**28.9**	**30.4**	**29.0**	**30.1**	**30.6**	**30.6**		*97.9*	*9.2*	*8.3*	*9.5*	*9.5*	*8.9*	*8.6*	*9.8*	*9.2*	*87.5*	*88.1*	*23.1*	*74.2*	*20.5*
**10.** **Avian orthoreovirus**	**36.5**	**36.9**	**48.7**	**35.0**	**34.8**	**38.0**	**50.3**	**49.7**	**30.8**		*9.2*	*8.3*	*9.8*	*9.8*	*9.2*	*8.9*	*9.8*	*9.5*	*87.2*	*87.8*	*23.1*	*73.3*	*21.4*
**11.** **Avian orthoreovirus (D20/99)**	**38.7**	**38.2**	**62.9**	**35.6**	**34.7**	**39.2**	**42.6**	**43.3**	**29.2**	**50.8**		*92.9*	*93.8*	*40.4*	*40.4*	*31.8*	*34.7*	*33.2*	*8.6*	*8.6*	*6.5*	*7.4*	*6.8*
**12.** **Avian orthoreovirus (PA/Turkey/22342/13)**	**40.1**	**39.8**	**74.7**	**37.4**	**38.7**	**40.2**	**47.2**	**46.6**	**29.9**	**50.4**	**64.2**		*88.4*	*39.5*	*39.8*	*32.0*	*34.4*	*33.8*	*7.7*	*7.7*	*5.9*	*6.5*	*6.2*
**13.** **Goose orthoreovirus (03G)**	**40.3**	**39.8**	**69.8**	**37.5**	**38.1**	**38.3**	**44.2**	**43.5**	**30.6**	**50.0**	**68.6**	**71.1**		*38.3*	*39.2*	*31.2*	*33.2*	*32.6*	*8.9*	*8.9*	*6.5*	*7.7*	*6.2*
**14.** **Mammalian orthoreovirus (3jin-1)**	**30.1**	**30.5**	**31.6**	**31.8**	**31.2**	**32.2**	**31.3**	**32.2**	**80.3**	**30.8**	**31.5**	**32.0**	**32.4**		*56.1*	*36.2*	*37.7*	*35.9*	*10.1*	*9.8*	*8.0*	*10.1*	*6.2*
**15.** **Mammalian orthoreovirus 3 (R124)**	**30.1**	**30.5**	**31.6**	**31.8**	**31.3**	**32.2**	**31.3**	**32.2**	**80.4**	**30.8**	**31.5**	**32.0**	**32.4**	**99.9**		*38.0*	*38.6*	*36.5*	*10.1*	*9.8*	*7.7*	*8.6*	*8.6*
**16.** **Mammalian orthoreovirus (729)**	**29.6**	**29.4**	**31.8**	**31.0**	**28.0**	**31.9**	**31.7**	**31.6**	**43.8**	**31.3**	**28.8**	**31.7**	**32.0**	**43.4**	**43.4**		*63.2*	*70.3*	*8.6*	*8.6*	*5.6*	*8.3*	*8.0*
**17.** **Ndelle virus**	**30.3**	**30.0**	**30.2**	**31.7**	**30.0**	**28.7**	**29.5**	**28.8**	**71.2**	**29.6**	**30.6**	**29.7**	**32.6**	**70.9**	**71.0**	**43.3**		*70.6*	*10.1*	*10.1*	*7.7*	*8.0*	*8.0*
**18.** **Porcine reovirus (SHR-A)**	**28.7**	**29.9**	**31.3**	**31.2**	**28.5**	**30.6**	**29.7**	**29.5**	**44.1**	**28.7**	**27.6**	**30.3**	**30.1**	**43.3**	**43.4**	**58.0**	**41.7**		*8.6*	*8.6*	*5.9*	*7.1*	*7.1*
**19.** **Mammalian Orthoreovirus (T3/Bat/Germany/342/08)**	**29.7**	**30.6**	**29.1**	**30.4**	**29.4**	**30.2**	**30.6**	**30.6**	**98.5**	**31.0**	**29.5**	**30.0**	**30.8**	**79.8**	**79.9**	**44.0**	**71.3**	**44.5**		*99.4*	*24.9*	*73.3*	*21.1*
**20.** **Melaka orthoreovirus**	**38.0**	**38.7**	**46.1**	**36.9**	**34.0**	**37.3**	**96.2**	**95.5**	**30.5**	**50.8**	**42.4**	**47.2**	**43.9**	**32.0**	**32.0**	**32.2**	**29.5**	**29.8**	**30.6**		*24.9*	*73.6*	*21.1*
**21.** **Nelson Bay orthoreovirus**	**37.4**	**38.8**	**45.6**	**36.5**	**34.9**	**37.3**	**83.3**	**83.4**	**31.5**	**49.4**	**41.4**	**46.8**	**43.0**	**31.4**	**31.4**	**32.2**	**30.5**	**30.6**	**31.4**	**83.9**		*23.1*	*50.7*
**22.** **Pulau reovirus**	**36.9**	**37.7**	**45.1**	**36.4**	**34.3**	**36.9**	**93.1**	**91.0**	**31.1**	**49.2**	**41.6**	**45.5**	**43.1**	**32.4**	**32.4**	**32.4**	**30.6**	**30.3**	**31.2**	**94.2**	**82.3**		*20.8*
**23.** **Tvarminne avian virus**	**35.3**	**35.6**	**48.9**	**35.4**	**32.6**	**39.6**	**50.8**	**49.6**	**30.3**	**61.7**	**47.4**	**49.0**	**48.5**	**31.1**	**31.1**	**32.5**	**30.6**	**30.2**	**31.0**	**50.1**	**48.6**	**49.3**	

**Table 4:** MAHLV genome characterization per segment.

Genome Segment		Gene							Gene Product					Public Sequence Database
		Size of Segment (nt)	5' End Penta-Nucleotide Sequence	3' End Penta-Nucleotide Sequence	5' UTR (nt)	3' UTR (nt)	Intergenic Region		Protein	Protein Size (aa)	Mw (kDa)	Proposed Function		Genbank Accession Numbers
L1		3950	5'-GGUCA	UCAUC-3'	14	51	-		Lambda A	1294	143.7	Core structural protein, binds dsRNA, NTPase, helicase		KU198602 to KU198621
L2		3925	5'-GGUCA	UCAUC-3'	16	33	-		Lambda C	1292	146.8	Guanylyl transferase, methyl transferase turret protein		
L3		3846	5'-GGUCA	UCAUC-3'	13	35	-		Lambda B	1265	142.7	Core protein, RNA-dependent RNA polymerase		
M1		2341	5'-GGUCA	UCAUC-3'	16	39	-		mu A	761	87.2	Core protein, transcription factor; ssRNA and dsRNA binding		
M2		2145	5'-GGUCA	UCAUC-3'	28	86	-		mu B	676	73.4	Outer capsid protein, membrane penetration during infection		
M3		2112	5'-GGUCA	UCAUC-3'	34	77	-		mu NS	666	75.6	Non-structural, virus inclusion		
S1		1322	5'-GGUCA	UCAUC-3'	13	58	-		Sigma A	416	47.8	Core protein, transcription factor; dsRNA binding; blocks interferon pathway		
S2		1282	5'-GGUCA	UCAUC-3'	31	69	-		Sigma B	393	44.7	Outer capsid protein		
S3		1209	5'-GGUCA	UCAUC-3'	27	63	-		Sigma NS	372	41.5	ssRNA binding; virus inclusion formation		
S4		1068	5'-GGUCA	UCAUC-3'	27	48	156		p14	125	13.9	Non-structural, membrane fusion (FAST)		
									p18	152	17.8	Non-structural, unknown function		
